# Clinical Evaluation of Sarcoidosis in Community Members with World Trade Center Dust Exposure

**DOI:** 10.3390/ijerph16071291

**Published:** 2019-04-10

**Authors:** Kerry M. Hena, Scarlett Murphy, Yian Zhang, Yongzhao Shao, Angeliki Kazeros, Joan Reibman

**Affiliations:** 1 Department of Medicine, New York University, New York, NY 10016, USA; scarlett.murphy@nyulangone.org (S.M.); angeliki.kazeros@nyulangone.org (A.K.); joan.reibman@nyulangone.org (J.R.); 2 Department of Population Health and Environmental Medicine, New York University, New York, NY 10016, USA; yian.zhang@nyulangone.org (Y.Z.); yongzhao.shao@nyulangone.org (Y.S.)

**Keywords:** sarcoidosis, World Trade Center (WTC), Scadding stage, lung function, severe lung disease, extrathoracic sarcoidosis, cardiac sarcoidosis

## Abstract

*Background*: Sarcoidosis is a granulomatous disease involving intrathoracic and extrathoracic organs. Genetic and environmental factors, such as exposure to World-Trade Center (WTC) dust after 9/11, may play a role in clinical presentation. Characterization of sarcoidosis in community members with exposure to the WTC dust can provide further insight into the relationship between environmental exposure and sarcoidosis. *Methods*: Patients with documented sarcoidosis were identified in the WTC Environmental Health Center (EHC), a treatment program for community members. Demographic and clinical data were collected from standardized questionnaires and chart review. Organ involvement was assessed with a standard instrument. *Results*: Among patients in the WTC EHC, 87 were identified with sarcoidosis after 9/11. Sarcoidosis cases were more likely African-American, local workers, and had more respiratory symptoms, compared with non-sarcoidosis WTC EHC patients. Many (46%) had ≥ Scadding stage 3 on chest imaging, and had reduced lung function measures. Extrathoracic involvement was identified in 33/87 (38%) with a diversity of organs involved. *Conclusions*: WTC-exposed sarcoidosis in community members is often characterized by severe pulmonary disease and a high rate of diverse extrathoracic involvement. Further analysis is required to characterize the course of disease progression or resolution.

## 1. Introduction

Sarcoidosis is a multisystem granulomatous disease of unknown etiology that is currently considered a genetically primed abnormal immune response to an antigenic exposure or trigger [1]. Rather than a single disease entity, sarcoidosis may be a constellation of “sarcoidoses,” with a characteristic pattern of organ involvement, depending upon the underlying genotype and triggering exposure. Exposure to the dust and fumes from the destruction of the World Trade Center (WTC) on 11 September 2001 (9/11) has been suggested to be one such trigger with sarcoidosis diagnoses described in WTC-exposed members of the Fire Department of the City of New York (FDNY), those involved in rescue and recovery efforts (responders), and community members (“survivors”) with WTC dust exposure [2,3,4,5,6,7]. Increased incidence rates have been reported for the firefighters and responders [2,3,6].

Whereas sarcoidosis can affect nearly all organs and tissues, intrathoracic involvement including hilar and mediastinal lymph nodes and/or lungs is the most common presentation [8,9]. However, extrathoracic involvement often distinguishes sarcoidosis from other granulomatous diseases and contributes to its diagnosis [9]. Indeed, in a large survey of sarcoidosis patients (A Case Control Etiologic Study of Sarcoidosis; ACCESS) 95% of the population had intrathoracic involvement and 50% extrathoracic involvement [8]. The presence of extrathoracic involvement was associated with age, race and sex [8]. In addition, differences in progression of intrathoracic and extrathoracic organ involvement were noted; intrathoracic involvement improved or remained stable whereas extrathoracic involvement typically increased over time [10,11]. Development of new organ involvement was more common in African-Americans [10]. The characteristics of sarcoidosis associated with WTC exposure, particularly those in community members, are less well described. A recent study of the clinical course of WTC-related sarcoidosis in the WTC-exposed Fire Department of the City of New York (FDNY) suggested a phenotype with a high rate of cardiac and rheumatologic (bone/joint) involvement, suggesting a unique response to their environmental exposure [7].

The WTC Environmental Health Center (WTC EHC) is a treatment program for self-referred community members, or “survivors,” with medical and mental health conditions secondary to acute exposure to the WTC dust on 9/11 (dust cloud) and/or chronic exposure to the WTC dust and fumes in their homes and/or workplaces over the ensuing days and months [12]. These community members include local residents, local workers, students, those involved in clean-up activities, and those who were passing by on 9/11. In contrast to the firefighter and responder populations, nearly 50% of the community members are women, and are of diverse race/ethnicity [13]. This heterogeneity and diversity of exposure to the WTC dust and fumes distinguishes them from the WTC-exposed responders, and their unique exposure sets them apart from the non-exposed general population. Characteristics of community members with sarcoidosis have been incompletely described. Exploration of their clinical presentation in comparison to other sarcoid variants may help us to better understand the relationship between exposure and sarcoidosis phenotype, providing further insight into sarcoidosis etiology. We now report a case series of community members in the WTC EHC with a diagnoses of post 9/11 sarcoidosis to further characterize the clinical presentation of sarcoidosis in this unique population. We describe similarities and differences between this population and other WTC and non-WTC sarcoidosis. 

## 2. Materials and Methods

### 2.1. Study Population

#### 2.1.1. Inclusion-Exclusion Criteria

Patients were included for review if they were enrolled in the Bellevue Hospital WTC EHC with a “certified” diagnosis as defined by the Centers of Disease Control (https://www.cdc.gov/wtc/handbook.html#certifications) and signed consent to have their data analyzed. By federal rules, WTC-exposed sarcoidosis cases allowed to enroll in the WTC EHC must have intrathoracic involvement presumed secondary to their relevant WTC exposure. The Institutional Review Board of New York University School of Medicine approved the research database (NCT00404898), and only data from patients who provided informed consent were used for analysis. Patients were included for analysis if they had biopsy proven sarcoidosis or chest imaging with characteristic findings of sarcoidosis defined as bilateral symmetrical mediastinal and/or hilar adenopathy, perilymphatic nodules, or fibrosis with bronchial distortion, at time of diagnosis or initial evaluation and without alternative etiology [9]. Signs or symptoms suggesting extrathoracic involvement such as confirmed uveitis or characteristic rash with concomitant elevated ACE level, were also used to establish a diagnosis [9]. Patients with a sarcoidosis diagnosis that predated 9/11 were excluded from analysis. 

#### 2.1.2. “Non-Sarcoidosis” Comparison Population

Study cases were compared to WTC exposed community members without sarcoidosis. In addition to WTC exposure, their entry into the WTC EHC required a physical complaint and diagnosis (e.g., lower or upper airway disease, GERD, other interstitial lung disease and/or cancer). 

### 2.2. Clinical Evaluation

Pathology reports were reviewed for all patients with available information to confirm histologic culture-negative, noncaseating granulomas. For those without a biopsy, imaging reports were reviewed to confirm characteristic findings. Information on demographic and clinical characteristics, including WTC and other exposures, were obtained from a standardized questionnaire completed upon entry into the WTC EHC [13]. Information in this questionnaire included direct exposures to the WTC dust clouds, potential for exposure in the home or workplace, and duration of exposure in the home or workplace prior to cleanup. The Modified Medical Research Council (mMRC) questionnaire was used to assess dyspnea. Additional clinical information obtained at entry into the WTC EHC included complete blood count, metabolic panel, and liver enzyme levels. Lung function measurements performed as routine screening were assessed. Chart reviews were performed to obtain additional information including CXR, computed tomography (CT) scans, ophthalmologic findings, EKG and advanced cardiac imaging. Additional diagnostic studies, e.g., serum angiotensin-converting enzyme (ACE) and serum vitamin D (25-hydroxy and 1,25 dihydroxy) levels obtained in the WTC EHC at time of entry into the program or during subsequent evaluation, were also reviewed. 

### 2.3. Assessment of Disease Severity and Organ Involvement

Sarcoidosis cases were staged for pulmonary disease by both CXR and/or CT scan using Scadding staging: stage 0: no adenopathy or infiltrates; stage 1: hilar and mediastinal adenopathy alone; stage 2: adenopathy and pulmonary infiltrates; stage 3: pulmonary infiltrates alone; and stage 4: pulmonary fibrosis [14]. Radiographic studies were selected for review based on temporality to disease onset with images used that were closest to date of diagnosis.

The World Association of Sarcoidosis and Other Granulomatous Diseases (WASOG) organ assessment instrument was used to categorize sarcoidosis involvement of each organ system at time of initial evaluation [15]. A finding of “highly probable” or “at least probable” was considered diagnostic for organ involvement. Positive findings on cardiac MRI (cMRI) were required to confirm a diagnosis of cardiac sarcoidosis, “at least probable” criteria by WASOG [15]. Other cardiac imaging modalities obtained after time of initial visit included electrocardiograms, echocardiograms, and continuous ambulatory ECG monitoring. These were reviewed for findings suggestive of possible cardiac involvement. Determination of ocular involvement required documentation of an ophthalmology visit in which fundoscopic findings confirming sarcoidosis were described. Patients with a brain MRI demonstrating characteristic abnormal enhancement after initial WTC clinic visit were identified as having neurologic involvement of sarcoidosis. Abnormal electromyography (EMG) findings were considered “suggestive but not diagnostic;” a field of “no consensus” in the WASOG organ assessment instrument [15]. Patients were identified as having cutaneous manifestations if biopsies showed pathognomonic granulomas and/or a dermatologist documented rashes with characteristic morphology “at least probable” or “highly probable” by WASOG [15]. Liver, spleen or gastrointestinal involvement was identified from available pathology, CT or MRI imaging. 

### 2.4. Statistics

Descriptive statistics were used to summarize the study cohort. We used count and percentage for categorical variables and median and IQR (Q1, Q3) for continuous variables. Univariate analyses were performed to compare the demographic characteristics, clinical characteristics, laboratory values and pulmonary function metrics between sarcoidosis patients and non-sarcoidosis patients and also between sarcoidosis patients who had lung disease at Scadding stage 1–2 and those who had stage 3–4 disease. The comparison tests include $\chi^{2}$ test for categorical variables and Mann-Whitney U test for continuous factors. Fisher exact test was performed in place of $\chi^{2}$ test if the expected count of a category was less than 5. All statistical analyses were accomplished using SAS 9.4 software (SAS Institute Inc., Gary, NC, USA). *p*-Values < 0.05 were considered statistically significant.

## 3. Results

### 3.1. Demographic Characteristics

Among 5849 community members at Bellevue Hospital enrolled between 17 August 2005 and 30 March 2018 with signed consents for analysis, 98 were identified with WTC-related sarcoidosis as per defined federal rules. Patients with a sarcoidosis diagnoses that predated 9/11 (*n* = 11) were excluded from analysis. This resulted in 87 individuals with sarcoidosis available for analysis. Biopsy-proven sarcoidosis was identified in 77, and an imaging diagnosis was obtained for 10. Sarcoidosis diagnosis occurred on average 7 years (SD = 4.5) after 9/11/2001 and 3 years (SD = 4.8) before enrollment in the WTC EHC, with mean time to enrollment being 10 years (SD = 4.4) after 9/11/2001. 

As far as the 5751 non-sarcoidosis community members, patients without lung function values (*n* = 607) were excluded to allow meaningful comparison of pulmonary physiology. For these non-sarcoidosis patients (*n* = 5144), time to enrollment in the WTC EHC occurred on average 9 years (SD = 3.8) after 9/11/2001.

Basic exposure and demographic characteristics are shown (Table 1). Many (51%) of the WTC-exposed community members with sarcoidosis were caught in the dust cloud on 9/11/2001, and most were local workers (73%). Almost half were female (48%) and the majority were never smokers (67%). Patients with sarcoidosis had a median age of 40 years on 9/11. Race/ethnicity was diverse with 44% reporting as black/African-American, 36% white and 18% Hispanic. We did not identify a statistically significant association between WTC dust cloud exposure and sarcoidosis status in WTC EHC. Those with sarcoidosis were more likely to be African-American and local workers compared with patients without sarcoidosis. 

### 3.2. Clinical Characteristics

Entry into the WTC EHC required a physical complaint and diagnosis, and most patients entered with respiratory symptoms and diagnoses consistent with asthma, chronic obstructive pulmonary disease or interstitial lung disease. Despite the frequency of these diagnoses, those with sarcoidosis were more likely to report dyspnea, cough and wheeze ≥ two times/week within 4 weeks of entry into the clinic, as well as a decreased exercise tolerance after 9/11 (Table 2). Many reported comorbid nasal drip or sinus congestion (57%) and heartburn (49%) while some endorsed rash (38%).

Lung function measurements including pre and post bronchodilator (bd) spirometry, lung volume and diffusion capacity measures were evaluated in patients with sarcoidosis compared with the non-sarcoidosis patients (Table 3). Sarcoidosis patients had a reduced FEV_1_ and pre-bronchodilator FEV_1_/FVC compared with non-sarcoidosis patients. Sarcoidosis patients were also characterized according to Scadding stage based on chest imaging. Thirty-six of 79 (46%) sarcoidosis patients with available chest imaging had severe lung disease defined as Scadding stage 3 or 4. Patients with more severe radiographic disease had reduced FEV_1_, FVC and D_L_CO compared with patients with milder radiographic changes (Table 3).

### 3.3. Organ Involvement

Thirty-three (38%) of the sarcoidosis patients had evidence of extrathoracic involvement: 19 (22%) had two organs involved, 10 (12%) had three organs involved, and 5 (6%) had ≥ four. Diverse organ involvement was identified; however, ocular involvement (15%) was the most common extrathoracic manifestation followed by skin (13%). Fewer had confirmed nervous system (3%) or cardiac (6%) involvement (Table 4). There were no significant differences in demographic or WTC exposure characteristics for isolated intrathoracic (*n* = 54) versus extrathoracic disease, severe lung disease (Scadding Stage 3 or 4; *n* = 36), or extent of extrathoracic organ involvement (3 or more organ systems involved; *n* = 15). Few patients (9/62; 15%) had elevated ACE levels. Median neutrophil-to-lymphocyte ratio (NLR) was 2.4, compared to a reported mean of 1.65 in an adult, non-geriatric, healthy population [17].

### 3.4. Cardiac Sarcoidosis: Screening and Diagnosis

Electrocardiograms were available for review in 75 individuals; 10 had abnormalities suggestive of potential cardiac sarcoidosis, including left or right bundle branch block, left anterior fascicular block or other interventricular conduction delay. Subsequent transthoracic echocardiogram was performed in 7/10 of these patients. The majority (86%) had normal left ventricular ejection fraction without any associated wall motion abnormalities. 

A total of 14 patients were referred for advanced cardiac imaging (either cMRI or PET). 6/14 were referred due to abnormal ECG, echocardiogram, and/or continuous ambulatory ECG monitoring; the remaining eight were referred due to concerning cardiac symptoms alone (e.g., atypical chest pain, palpitations, dizziness/syncope). Cardiac involvement was confirmed in 5/14 patients, one with a supporting myocardial biopsy and two with an unremarkable screening study. Many patients were awaiting advanced cardiac imaging at the time of this publication, including 7 patients with an abnormal EKG and 6 with abnormal continuous ambulatory ECG monitoring. 

## 4. Discussion

We described the clinical characteristics of a heterogeneous cohort of WTC-exposed community members, or “survivors,” with post-9/11 sarcoidosis. The demographic characteristics of the WTC EHC population with sarcoidosis differed from the FDNY, with many more patients who were female or who identified as African American or Hispanic. These characteristics were more similar to the general population described in ACCESS and the cohort from Medical University of South Carolina (MUSC) [8,10,11].

By definition, WTC-exposed sarcoidosis cases must have intrathoracic involvement (100%). However, the WTC-exposed survivors with sarcoidosis differed from other sarcoidosis variants—the WTC-exposed FDNY cohort as well as previously reported non-exposed general population(s)—in terms of severity of their intrathoracic disease [7,8,10,11]. Forty-six percent had Scadding stage 3 or 4 on initial evaluation chest imaging, indicating advanced pulmonary involvement, which was associated with reduced lung function measures. This frequency of severe disease is higher than that described in the initial ACCESS study (15% with stage 3 or 4) and even greater than that described in the FDNY cohort (7%) [7,8]. Importantly, the MUSC study, which evaluated the last available radiograph allowing for progression of Scadding stage, still described lower rates (27%) of stage 3 or 4 disease than that identified in the WTC EHC cohort (46%). The severity of intrathoracic disease is consistent with the respiratory symptoms reported at time of enrollment (Table 2). However, it is unknown whether our cohort represents those with more symptomatic, severe disease rather than sarcoidosis all-comers given the distinct self-referral entry into the WTC-EHC. 

The frequency of extrathoracic involvement (38%) was slightly less than that reported in ACCESS (50%) [8]. However, the extrathoracic involvement in the WTC-EHC was distinguished by its variety and extent of extrathoracic organs involved. Similar to general population studies with comparable race/ethnic distribution, ocular and skin involvement were most common [8,10,11]. This is in contrast to the predominantly Caucasian male FDNY cohort, for which eye and skin involvement were relatively rare (5% and 2% respectively) [7]. Rare, but unique organ involvement included thyroid (1%), gastrointestinal (2%) and the appendix (1%). The extent of organ involvement (17% with three or more organs involved), was similar to that reported in the general population (20%) [8]. However, unlike the ACCESS and MUSC studies where African Americans tended to have more organs involved, we detected no significant differences in demographic variables for intrathoracic versus extrathoracic disease or extent of extrathoracic organ involvement [8,10,11].

Cardiac sarcoidosis remains a challenging diagnosis with current guidelines recommending advance cardiac imaging only in patients with symptoms, abnormal (ECG), or abnormal echocardiogram [18,19]. However, screening modalities have limited sensitivity; a study on predictors of cardiac sarcoidosis cited a sensitivity of 58% for ECGs and 62% for echocardiograms [20]. While continuous ambulatory ECG monitoring for 24 hours or more has higher sensitivity (89%), it has low specificity (21%) [20]. The rate of confirmed cardiac sarcoidosis (6%) in the survivor cohort is consistent with that of non-WTC general population studies, with clinically evident cardiac sarcoidosis manifested by conduction abnormalities, ventricular arrhythmias, and/or heart failure evident in only 2–7% of sarcoidosis patients [8,10,11]. Although cardiac sarcoidosis was clinically detected in 16% (19/57) of the WTC-exposed FDNY cohort at follow-up, this may have been due to increased screening in relatively asymptomatic patients [7]. A lower threshold for advanced cardiac imaging in the survivor population might result in higher rates of detection, beyond those already awaiting confirmatory diagnostics. 

This study has several limitations. Complete analyses of all patients were not possible due to missing data. Many patients referred to the WTC EHC also receive care at outside institutions, including some critical components of sarcoidosis screening for extrathoracic disease. Thus data from other institutions may not have been captured by our study, leading to potential underreporting of extrathoracic organ involvement. Furthermore, although a standardized approach to diagnosis and evaluation was recommended at the WTC EHC, diagnostic studies were varied. Patients shared a common WTC exposure; however, the degree and extent of that exposure, including differences among the WTC dust characteristics, is known to vary, complicating the environmental exposure analysis. Patients had ranging dates of initial sarcoidosis diagnosis. Given the tendency of sarcoidosis organ involvement to increase per year, it is likely that patients in our cohort with more recent dates of diagnosis will continue to develop extrathoracic involvement as time progresses. In addition, longitudinal analysis was confounded by irregular follow-up by many patients, and visit incongruity made it difficult to compare number of organs involved per equivalent time period. Additional analyses are needed to further characterize clinical course. 

## 5. Conclusions

Our data suggest a WTC-exposed sarcoidosis phenotype in community members or “survivors” often characterized by severe pulmonary disease and diverse extrathoracic involvement. These characteristics differ from those described for the FDNY. Similarities to general population studies are likely secondary to comparable gender and race/ethnicity distribution with differences in severity of pulmonary disease and rare organ involvement potentially explained by WTC exposure. Further analysis is required to characterize the course of disease progression or resolution.

## Figures and Tables

**Table 1:** Basic demographic characteristics.

Characteristic	Non-Sarcoidosis (*N* = 5144)	Sarcoidosis (*N* = 87)	*p*-Value
Age on 9/11/2001, *yr*, median (Q1, Q3)	43 (35, 50)	40 (33, 45)	0.015 *
Age on Initial Visit, *yr*, median (Q1, Q3)	59.7 (52, 67)	57.5 (51, 62)	0.017 *
Gender, *n* (%)			0.8
Female	2551 (50)	42 (48)	
Male	2593 (50)	45 (52)	
Race/Ethnicity, *n* (%)			<0.0001 ***
Asian	427 (8)	2 (2)	
Hispanic	1483 (29)	15 (18)	
NH-Black	942 (19)	38 (44)	
NH-White	2176 (43)	31 (36)	
Native American	11 (0.2)	-	
Other	51 (1)	-	
Ever smoke cigarettes, *n* (%)			0.2
Yes	2032 (40)	29 (33)	
No	3087 (60)	58 (67)	
WTC Exposure Category, *n* (%)			<0.0001 ***
Local worker	2751 (53)	63 (73)	
Resident	1163 (23)	8 (9)	
Rescue/Recovery/Other	603 (12)	15 (17)	
Clean-up worker	613 (12)	1 (1)	
WTC Dust Cloud Exposure, *n* (%)			0.6
Yes	2711 (53)	44 (51)	
No	2388 (47)	43 (49)	

*p*-Value: *** < 0.0001; * < 0.05.

**Table 2:** Respiratory symptoms at time of enrollment.

Symptom, *n* (%)	Non-Sarcoidosis (*N* = 5144)	Sarcoidosis (*N* = 87)	*p*-Value
Cough	3278 (64)	67 (78)	0.009 **
Wheezing	2608 (51)	58 (67)	0.004 **
Dyspnea with exercise	3929 (77)	76 (89)	0.009 **
Dyspnea at rest	1731 (34)	49 (56)	<0.0001 ***
MMRC			0.03 *
<3	3509 (79)	56 (69)	
≥3	928 (21)	25 (31)	

*p*-Value: *** < 0.0001; ** < 0.01; * < 0.05.

**Table 3:** Lung function: Stage 1–2 vs. Stage 3–4 pulmonary sarcoidosis.

	Non-Sarcoidosis (*N* = 5144)	Sarcoidosis (*N* = 79)		Non-Sarcoidosis vs. Sarcoidosis *p*-Value	Stage 1–2 vs. Stage 3–4 *p*-Value
		Lung Disease Stage 1–2 (*N* = 43)	Lung Disease Stage 3–4 (*N* = 36)		
FEV_1_, *L*, median (%^†^)					
Pre bd	2.69 (90)	2.86 (88)	2.10 (75)	0.03 *	0.002 **
Post bd	2.80 (94)	3.01 (89)	2.06 (77)	0.02 *	0.0004 **
FVC, *L*, median (%^†^)					
Pre bd	3.51 (92)	3.55 (92)	3.02 (83)	0.25	0.03 *
Post bd	3.56 (93)	3.68 (89)	2.78 (81)	0.13	0.008 **
FEV_1_/FVC, %^†^					
Pre bd	78	76	75	0.04 *	0.65
Post bd	80	79	79	0.27	0.42
TLC, *L*, median (%)	5.15 (91)	4.83 (90)	4.39 (67)	0.22	0.22
DLCO, *mmHg*, median (%)	19.15 (87)	19.50 (84)	15.10 (60)	0.11	0.013 *

FEV_1_: forced expiratory volume in one second; FVC: forced vital capacity; TLC: total lung capacity; DLCO: diffusing capacity of the lungs for carbon monoxide; bd: bronchodilator; %^†^: National Health and Nutrition Examination Survey (NHANES) III reference values [16]; *p*-Value: *** < 0.0001; ** < 0.01; * < 0.05.

**Table 4:** Organ involvement.

Organ System, *n* (%)		Sarcoidosis (*N* = 87)
Intrathoracic *	Stage 1	18 (23)
	Stage 2	25 (32)
	Stage 3	17 (22)
	Stage 4	19 (24)
Cardiac		5 (6)
Eyes		13 (15)
ENT		3 (3)
Bone-Joints		1 (1)
Bone marrow		2 (2)
Skin		11 (13)
Nervous System		3 (3)
Liver		3 (3)
Spleen		3 (3)
Kidney		1 (1)
Calcium		3 (3)
Extrathoracic lymph nodes		2 (2)
Thyroid		1 (1)
Gastrointestinal		2 (2)
Appendix		1 (1)

* All with certified pulmonary involvement; eight without readily available chest imaging for review (*n* = 79).
